# Predictors and Causes of Implicit Rationing of Nursing Care: Lessons From the COVID‐19 Pandemic Crisis in Iran: A Cross‐Sectional Study

**DOI:** 10.1155/nrp/7362186

**Published:** 2026-09-15

**Authors:** Zahra Tagharrobi, Tayebeh Moradi, Khadijeh Sharifi, Negin Masoudi Alavi

**Affiliations:** ^1^ Trauma Nursing Research Center, Kashan University of Medical Sciences, Kashan, Iran, kaums.ac.ir; ^2^ Department of Nursing, Kas.C., Islamic Azad University, Kashan, Iran, azad.ac.ir

**Keywords:** COVID-19 pandemic, Iran, nurse, predictors, rationing of nursing care

## Abstract

**Background:**

Rationing of nursing care (RONC) represents a significant challenge for healthcare systems, particularly during pandemics. Effective management of RONC requires thorough examination and identification of the contributing factors within crisis situations. This study aimed to investigate the predictors and causes of implicit RONC amidst the COVID‐19 pandemic in Iran.

**Materials and Methods:**

A cross‐sectional study was conducted in 2022, involving 200 nurses selected via stratified random sampling from COVID‐19‐designated hospitals in Kashan city. Data were collected using the Persian version of the Perceived Implicit Rationing of Nursing Care (P‐PIRNCA) Questionnaire, the MISSCARE survey, and a questionnaire detailing potential predictors of implicit rationing. Data were analyzed using SPSS version 22, employing one‐way ANOVA, Independent *t*‐test, Kruskal–Wallis test, Pearson and Spearman–Brown correlation coefficients, and multiple linear regression.

**Results:**

The mean score for the RONC was 36.02 ± 19.79 (95% CI: 33.28–38.77). Interest in the nursing profession, second job, number of children, and monthly income were reported to be significant in the rationing model (*p* < 0.0001, *R* ^2^ = 0.206). Interest in the nursing profession emerged as the most significant predictor, accounting for 10.5% of the explained variance in nursing care rationing scores. Communication, human, and material factors explained 85.3%, 10.2%, and 4.5% of the variance in importance scores of reasons for missed care, respectively.

**Conclusion:**

The RONC was not high at the peak of the pandemic. Selecting nurses from among those interested in the profession, those without a second job, and those with more children, as well as paying attention to professional relationships, can lead to adjustments in the rationing of care during the difficult conditions of the pandemic crisis.

## 1. Introduction

The COVID‐19 pandemic has posed an unprecedented challenge to health systems worldwide. One of the major challenges during the COVID‐19 pandemic was the implicit rationing of nursing care (IRONC), which refers to the systematic omission or delay of necessary nursing interventions when time, staffing, or material resources are insufficient [1, 2].

In the literature, the broader term RONC refers to situations in which nurses intentionally or unintentionally fail to perform essential patient care tasks due to insufficient time, inadequate staffing levels, or inappropriate skill mix [3]. RONC is the process of controlling or restricting the distribution of care among patients according to conditions related to nurses, patients, organizations, and care that can lead to rationed or missed nursing care [3, 4].

RONC occurs when the available resources and conditions are insufficient to provide comprehensive care. Under such circumstances, nurses may be unable to deliver care in accordance with professional standards [4, 5]. In this context, rationing refers to the failure to complete all required nursing activities during a shift [6]. IRONC specifically captures the unspoken or unconscious dimension of care rationing, that is, nurses’ implicit decisions regarding which interventions to perform, postpone, or omit, without formal guidelines or explicit organizational directives [3, 7].

Empirical studies report that the prevalence of RONC ranges from 55% to 98% worldwide [1, 8], and it is considered an accurate indicator of patient safety and the quality of nursing care [1]. Failure to provide complete care, or partial implementation of care, may lead to errors or complications for the patient, endangering their life [3, 4]. Evidence indicates that inadequate nursing care is associated with adverse outcomes such as increased mortality, hospital‐acquired infections, patient falls, pressure ulcers, and reduced quality of care [3, 4, 6]. The consequences of RONC may be patient‐related (e.g., pressure ulcers), nurse‐related (e.g., moral distress), or organizational (e.g., increased length of hospital stay) [4, 9].

The COVID‐19 crisis intensified conditions conducive to nursing care rationing. Increased workload, patient surge, inadequate staffing, and emotional strain disrupted nurses’ capacity to deliver comprehensive care [1, 9, 10]. In addition to heightened care demands, nurses faced unfamiliar and crisis‐driven work environments resulting from changes in hospital layouts, exposure to disease, and interdepartmental transfers. They also experienced elevated levels of anxiety, depression, and burnout during the pandemic [11, 12]. Previous studies have indicated that nurse shortages, poor working conditions, negative emotions, and imbalanced nurse–patient ratios can increase RONC occurrences during a pandemic, further reducing the quality of care [13, 14]. For example, a study conducted in Iran by Hosseini et al. reported that an increased number of patients, staff shortages, extended working hours, reassignment to new roles, and high levels of chronic stress during the pandemic disrupted the nursing care process and increased RONC [15]. Other studies among Iranian nurses have also suggested that crisis‐related stressors during the COVID‐19 pandemic adversely affected nurses’ comfort and, consequently, nursing care quality [16]. Studies from other countries have also reported high levels of missed care and rationing of care during the pandemic. In the study by Gurková et al., missed care was more frequent during the pandemic [17]. Another study reported an increase in missed nursing care during the pandemic [18].

In contrast, some studies have reported different findings. Von Vogelsang et al. observed little difference in the level of missed care before and during the pandemic. Most participants reported maintaining good quality of care and patient safety due to stable registered nurse–to–patient ratios and effective managerial support [19]. Another study found that although the pandemic significantly affected ICU personnel, it only slightly influenced elements of missed nursing care [20].

Studies also vary in explaining the causes of RONC. Some have identified human factors and resource shortages as the primary contributors to care rationing and missed nursing care [21–23]. In contrast, others have emphasized communication problems as having a greater impact than human and material resource constraints [24].

Given this contradictory finding worldwide and the limited evidence from Middle‐Eastern contexts, particularly during crisis situations [25], further research is warranted to clarify the underlying causes and predictors of IRONC during health crises. Considering these gaps and inconsistencies in the literature, this study aimed to identify the reasons and predictors of IRONC among nurses working in Iranian hospitals during the COVID‐19 pandemic.

## 2. Methods

### 2.1. Study Design and Population

This cross‐sectional study was conducted in 2022 at COVID‐19‐designated hospitals affiliated with Kashan University of Medical Sciences, including Kashan Shahid Beheshti Hospital and Seyed Al‐Shohada Hospital in Aran and Bidgol.

The study population consisted of nurses working in inpatient wards dedicated to patients with COVID‐19 who met the study inclusion criteria. The inclusion criteria were [1] holding a university degree in nursing; [2] having at least three months of clinical work experience; [3] working for a minimum of two weeks in wards dedicated to COVID‐19 patients; and [4] self‐reported absence of acute psychological disorders that could impair their ability to provide accurate self‐assessment during the study period.

Exclusion criteria included refusal to provide written informed consent, withdrawal of consent at any stage of the study, or failure to complete more than 10% of the total items across all administered instruments.

### 2.2. Sample Size and Sampling Method

This study aimed to assess two primary objectives: Perceived Implicit Rationing of Nursing Care (PIRNCA) and the importance of its causes (Part B of the Kalisch Missed Nursing Care Survey).

To ensure adequate statistical power for both study objectives, the required sample size was estimated using the standard formula for a population mean with a known standard deviation: $n={\left(Z_{12-\alpha /}\times \sigma /d\right)}^{2}$, assuming a 95% confidence level (*Z* = 1.96).

The desired precision (d) was defined as 0.14 of the respective standard deviation (i.e., a relative precision of 14%), consistent with previous literature. Based on reported standard deviations, 12.59 for missed nursing care (Vatankhah et al., [26]) and 0.44 for the importance of causes (Karimi et al., [27]), two estimates of *n* were obtained. The final required sample size was set to the larger of the two estimates, resulting in a target sample of *N* = 200 participants.

Sampling was performed using a stratified random sampling method based on the hospital ward. Initially, clinical departments dedicated to COVID‐19 care—including internal medicine wards and intensive care units (ICUs) at Shahid Beheshti Hospital (Kashan) and Seyed Al‐Shohada Hospital (Aran and Bidgol)—were identified. A list of eligible nurses in these wards was then prepared. Participants were randomly selected proportionately to the number of qualified nurses in each ward to achieve the required sample size.

### 2.3. Data Collection Procedures

Both paper‐based and online versions of the questionnaires and consent forms were prepared. Selected nurses were contacted by telephone to receive information about participation and to give informed consent. Respondents completed the survey according to their preferred mode (paper‐based or online). In cases where the questionnaire was not completed within 24 h, a follow‐up reminder was sent to the nurse. The nurse had the right to withdraw. In the event of noncompliance or withdrawal from the study, or if the instruments were not completed, the nurse would be excluded from the study, and another nurse from the same department would be selected by simple random sampling to replace them.

### 2.4. Tools

Three instruments were used for data collection: the Probable Predictors of Implicit Rationing of Nursing Care, the Persian version of the PIRNCA [28], and Part B of the Kalisch Missed Nursing Care Survey [29]. The instruments were completed in a self‐report format, either on paper or online, according to the participants’ preference.

1. The Probable Predictors of Implicit Rationing of Nursing Care:
 This tool collects demographic information, such as age, gender, marital status, education level, nativeness, spouse’s occupation, number of children, family income, and housing status, as well as occupational information, including nursing work experience, current employment status, position, predominant shift type, department of employment, work experience in current ward (COVID‐19 ward), having overtime, average weekly working hours, monthly salary, whether having a second job, interest in nursing profession, job satisfaction, and teamwork satisfaction in the COVID‐19 ward. The tool’s qualitative content validity was confirmed by a number of nursing faculty members.
2. PIRNCA
 Moradi et al. translated and psychometrically evaluated the PIRNCA questionnaire in Iran in 2022. The Persian version of this questionnaire (P‐PIRNCA) consists of 31 items in 5 dimensions. The first dimension, patient examinations and order execution, contains eight questions, including questions 21, 20, 19, 13, 12, 11, 9, and 6; the second dimension, documentation/support, contains seven questions, including 31, 30, 29, 28, 27, 17, and 14; the third dimension, communication and personal hygiene, contains eight questions, including 26, 25, 24, 23, 22, 3, 2, and 1; the fourth dimension, client comfort and convenience, contains eight questions, including questions 10, 8, 7, 5, and 4; and, finally, the fifth dimension, status follow‐up and client education, contains three questions, including questions 15, 16, and 18. This tool considers four responses (never, rarely, sometimes, and often) for each item, with scores ranging from 0 to 3. The questionnaire also includes an option called “not required.” The total score obtained from the tool ranges from 0 to 93. Since “not required” is an option for each item, a maximum score percentage is defined for the total score of the instrument as well as for each domain. This percentage is calculated by subtracting the minimum possible score from the maximum possible score, then dividing the total score by this difference. In other words, the score is determined on a scale from zero to 100. In this tool, a higher score indicates more rationing. The Persian version’s content and construct validity have been examined and confirmed. The estimated Cronbach’s alpha and test–retest intraclass correlation coefficients (ICC) are above 0.9 [28].
3. Part B of the Kalisch Missed Nursing Care Survey
 Kalisch developed the Missed Nursing Care Survey (MISSCARE Survey) in 2006 and psychometrically evaluated it in 2009 [29]. Rahimi et al. translated and psychometrically evaluated this tool in Iran, presenting it as the MISSCARE‐Persian Survey. This questionnaire has two parts: Part A is about neglected nursing care, and Part B is about the reasons for it. The present study used Part B of the instrument. This instrument contains 17 items across three categories of organizational, material, and communicational dimensions. The importance of each item (cause) has a score between 1 and 4, and the importance of each dimension and all causes is measured by summing the scores of the related items. The total score of the instrument ranges from 17 to 68, with higher scores indicating a greater perceived importance of the causes of missed care. The Cronbach’s alpha coefficient for the second part of the instrument was estimated to be 0.901 [30]. The Cronbach’s alpha of the instrument was estimated to be 0.87 in the present study.

### 2.5. Ethical Considerations

Permission was obtained from the Research Council (No. 40092) and the Ethics Committee of Kashan University of Medical Sciences (Code: KAUMS.NUHEPM.REC.1400.040.IR). Verbal and written consent was obtained from the nurses. The purpose of the study was explained to the participants, who were also assured that their information would be kept confidential. The nurses were informed that they had the right to withdraw from further cooperation.

### 2.6. Statistical Analysis

The collected data were analyzed using SPSS Version 22. The Nursing Care Rationing Score was determined on a scale of 0–100, and its range was estimated with 95% confidence. This study was reported in line with the Strengthening the Reporting of Observational Studies in Epidemiology (STROBE) guidelines [31]. To examine the normality of the quantitative data, the indices of Kurtosis and skewness were used (a range of ±2 was considered to be normal). To prioritize the components of the nursing care rationing questionnaire and assess the relative importance of the causes—that is, the contribution of each instrument dimension in explaining the variance in the implicit nursing care rationing score or the perceived importance of the causes—stepwise linear regression analysis was employed. The analysis was conducted in two stages to identify the predictors. In the first stage, each factor was examined separately using univariate analysis. To examine the relationship between the rationing score and categorical variables, independent *t*‐test, analysis of variance (ANOVA), or Kruskal–Wallis test was used. To examine the relationship between the rationing score and quantitative variables, the Pearson or Spearman correlation coefficient was used. To more accurately examine the relationships and contributions of each factor to the rationing score, stepwise multiple linear regression analysis was performed. At this stage, variables with a *p* value less than 0.2 in the univariate analysis were entered into the model as independent variables, with the rationing score serving as the dependent variable. A significance level of *p* < 0.05 was considered statistically significant in all analyses.

## 3. Results

A total of 235 individuals were invited to participate in the study, of whom 200 agreed to participate, completed the questionnaires in full, and were included in the data analysis. The sample was drawn from two distinct hospital settings: 85% of the participants were recruited from Shahid Beheshti Hospital in Kashan, and the remaining 15% were sourced from Seyed Al‐Shohada Hospital in Aran and Bidgol. The majority of nurses (66.5%) were allocated to internal wards dedicated to COVID‐19 patients, while the remaining 33.5% were assigned to ICUs for COVID‐19 care (see Table 1 for demographic breakdown).

**TABLE 1:** Demographic characteristics of the assessed clinical nurses, Kashan, 2022 (*n* = 200).

Qualitative V			
Variables	Category		*n* (%)
Gender	Male		41 (20.5)
	Female		159 (79.5)
Marital status	Single		25 (12.5)
	Married		175 (87.5)
Education level	Bachelor’s		181 (90.5)
	Associate degree		19 (9.5)
Native of Kashan	Yes		189 (94.5)
	No		11 (5.5)
Spouse’s occupation	Employee		54 (27)
	Freelancer		98 (49)
	Other		19 (9.5)
	No response		29 (14.5)
Family income	Adequate		5 (2.5)
	Moderate		138 (69)
	Inadequate		57 (28.5)
Housing status	Ownership		154 (77)
	Rent		44 (22)
	Company housing		2 (1)
Hospital	Shahid Beheshti Hospital		170 (85)
	Seyyed Al‐Shohada Hospital		30 (15)
Employment status	Official		107 (53.5)
	Temporary‐to‐official		50 (25)
	Contractual		12 (6)
	Conscription law		6 (3)
	Other		25 (12.5)
Work position	Rotating nurse		186 (93)
	Staff Nurse		14 (7)
Predominant shift type	Morning		36 (18)
	Evening		16 (8)
	Night		6 (3)
	Rotating		142 (71)
Department of employment	Internal (COVID‐19)		133 (66.5)
	Intensive care (COVID‐19)		67 (33.5)
Having overtime	Yes		175 (87.5)
	No		25 (12.5)
Second job (non‐nursing activities)	Yes		16 (8)
	No		184 (92)

**Quantitative V**			
**Variable**	**Mean ± SD**	**Min**	**Max**

Age (year)	31.60 ± 6.41	22	49
Number of children	0.87 ± 0.88	0	3
Nursing experience (year)	8.36 ± 8.04	0.5	76
Work experience in the current ward (month)	3.74 ± 3.22	0.5	18
Overtime per month (hour)	46.99 ± 10.00	25	84
Monthly salary (thousand tomans)	8461.76 ± 1277.39	6000	11,000
Interest in nursing profession (0–10)	6.90 ± 2.53	0	10
Job satisfaction (0–10)	5.59 ± 2.82	0	10
Teamwork satisfaction (0–10)	6.48 ± 2.62	0	10

The implicit rationing score among participants, on a scale of 0–100, was 36.02 ± 19.79, with a 95% confidence interval ranging from 33.28 to 38.77. The score of the first dimension, patient examinations and order execution, was 30.196 ± 22.365 (95% CI: 26.873–33.518); the score of the second dimension, documentation/support, was 40.773 ± 24.537 (95% CI: 37.304–44.243); the score of the third dimension, patient communication and personal hygiene, was 37.337 ± 19.519 (95% CI: 34.255–40.418); the score of the fourth dimension, client comfort and convenience, was 26.755 ± 17.090 (95% CI: 24.312–29.197); and finally, the score of the fifth dimension, status follow‐up and client education, was 20.033 ± 16.170 (95% CI: 17.781–22.285). In prioritizing the dimensions of the nursing care rationing questionnaire, stepwise linear regression indicated that the contributions of each dimension to explaining the variance in the rationing score were 72.2%, 12.9%, 2.9%, 2.7%, and 1.3%, respectively.

Univariate analysis revealed that gender, family income, predominant shift, employment in non‐nursing activities as a second job, age, number of children, interest in the nursing profession, job satisfaction, and teamwork satisfaction in the COVID‐19 ward were significantly associated with the nursing care rationing score (Table 2).

**TABLE 2:** IRONC score based on personal and occupational variables among Iranian nurses, (*n* = 200).

A: Maximum percentage of IRONC score based on categorized variables						
Variable	Categories	IRONC score mean ± SD	*t*/*F*/*χ* ^2^	*p* value		
Gender	Female	47.07 ± 23.32	−3.559^a^	0.001		
	Male	33.17 ± 17.77				
Education level	Bachelor’s	36.26 ± 20.05	−0.545^a^	0.587		
	Associate degree	33.66 ± 17.35				
Marital status	Single	37.94 ± 18.52	0.519^a^	0.604		
	Married	35.74 ± 20.00				
Spouse occupation	Employee	34.99 ± 18.34	2.144^b^	0.120		
	Freelancer	33.39 ± 19.96				
	Other	43.49 ± 19.84				
Department of employment	Internal (COVID‐19)	34.59 ± 20.01	1.438^b^	0.152		
	Intensive care (COVID‐19)	38.85 ± 19.18				
Native of Kashan	Yes	35.98 ± 19.99	0.119^a^	0.905		
	No	36.71 ± 16.73				
Family income	Adequate (A)	12.25 ± 0.96	5.839^c^	Pairwise comparison^d^		
	Moderate (B)	34.86 ± 17.75		0.003	A, B A, C B, C	> 0.0001 > 0.0001 0.191
	Inadequate (C)	40.89 ± 23.30				
Housing status	Ownership	35.48 ± 19.77	2.155^b^	0.119		
	Rent	39.04 ± 19.57				
	Company housing	11.29 ± 0.76				
Employment status	Official	34.28 ± 19.13	5.888^e^	0.208		
	Temporary‐to‐official	40.93 ± 20.50				
	Contractual	33.01 ± 28.47				
	Conscription law	31.17 ± 28.56				
	Other	36.24 ± 12.49				
Position	Rotating nurse	36.60 ± 19.85	1.532^a^	0.127		
	Staff Nurse	28.23 ± 17.79				
Predominant shift type	Morning (A)	26.22 ± 14.86	20.318^e^	Pairwise comparison^f^		
	Evening (B)	47.84 ± 16.29		A, B	> 0/0001	
	Night (C)	47.86 ± 24.26		A, C	0/078	
	Rotating (D)	36.67 ± 20.05		A, D	0/009	
				B, C	1	
				B, D	0/093	
				C, D	1	
Having overtime	Yes	35.43 ± 18.64	0.625^a^	0.532		
	No	40.11 ± 26.61				
Hospital	Shahid Beheshti Hospital	37.50 ± 19.89	1.106^a^	0.270		
	Seyyed Al‐Shohada Hospital	27.62 ± 17.21				
Second job (non‐nursing activities)	Yes	56.02 ± 25.30	3.360^a^	0.004		
	No	34.28 ± 18.31				

**B: Correlation of the IRONC score with quantitative variables**						
**Variable**	***r* values**	***p* value**				

Age	−0.151^g^	0.032				
Number of children	−0.174^g^	0.014				
Work experience in the current ward (months)	−0.066^g^	0.357				
Nursing experience (year)	−0.057^h^	0.423				
Overtime per month (hours)	0.078^g^	0.27				
Average working hours per week	−0.132^g^	0.096				
Monthly salary (thousand tomans)	0.126^g^	0.101				
Interest in nursing profession (0–10)	−0.324^g^	> 0.0001				
Job satisfaction (0–10)	−0.285^g^	> 0.0001				
Teamwork satisfaction (0–10)	−0.141^g^	0.047				

^a^Independent *t*–test.

^b^ANOVA.

^c^Welch statistic/ANOVA.

^d^Post hoc (Games–Howell).

^e^Test statistic/Kruskal–Wallis.

^f^Adjusted by the Bonferroni correction.

^g^Pearson coefficient.

^h^Spearman–Brown coefficient.

To more accurately examine the relationship between the independent variables and the nursing care rationing score, stepwise multiple linear regression was performed. The results showed that the variables of interest in the nursing profession, second job (non‐nursing activity), number of children, and monthly salary were significant predictors in the model (*F* = 12.636, *p* < 0.0001). Collectively, these variables explained 20.6% of the observed variance in the nursing care rationing score (*R* ^2^ = 0.206). The largest contribution was related to the variable of interest in the nursing profession, which alone explained 10.5% of the variance. Monthly salary and having a second job (non‐nursing activity) had a positive effect on the rationing score. Interest in the nursing profession and the number of children were associated with lower levels of rationing (Table 3).

**TABLE 3:** The results of multiple linear regression to determine the predictors of IRONC among Iranian nurses.

Model	*R* ^2^	*B*	95% CI B		SE	Beta	*t* value	*p* value
			Lower	Upper				
Constant	—	51.458	23.02	79.85	14.400	—	3.573	> 0.0001
Interest in nursing	0.105	−2.11	−3.18	−1.03	0.543	−0.271	−3.884	> 0.0001
Second job (non‐nursing activities)	0.045	13.877	23.78	3.97	5.023	0.191	2.763	0.006
Number of children	0.020	−4.605	−7.615	−1.596	1.526	−0.207	−3.018	0.003
Monthly salary	0.036	0.004	0.001	0.006	0.001	0.209	2.961	0.003

*Note:* Model summary: *R* ^2^ = 0.206, *F* = 12.635, *p* < 0.0001.

The mean importance score for the reasons behind RONC, on a scale of 17–68, was 55.65 ± 7.15, with a 95% confidence interval ranging from 54.66 to 56.64 in the study population.

The importance of each reason for missed nursing care is shown in Table 4. Table 4 shows the importance scores for the reasons behind RONC. On a scale of 3–12, material factors had a mean score of 9.73 ± 1.92; on a scale of 6–24, human factors scored 21.85 ± 2.18; and on a scale of 8–32, communication factors scored 24.05 ± 4.96 (Table 4).

**TABLE 4:** Causes of missed nursing care by importance from the perspective of nurses.

Area	Reason	Significant *n* (%)	Moderate *n* (%)	Minor *n* (%)	Not a reason *n* (%)	Item score Mean ± SD (scale of 1–4)	Area score Mean ± SD
Human factors On a scale of 6–24	Inadequate number of staff	154 (77)	39 (19.5)	7 (3.5)	0	3.74 ± 0.516	21.85 ± 2.18
	Urgent patient situation (e.g., a patient’s condition worsening)	118 (59)	59 (29.5)	23 (11.5)	0	3.48 ± 0.049	
	Unexpected rise in patient volume and/or acuity on the unit	163 (81.5)	32 (16)	5 (2.5)	0	3.79 ± 0.466	
	Inadequate number of assistive and/or clerical personnel	145 (72.5)	41 (20.5)	14 (7)	0	3.66 ± 0.606	
	Unbalanced patient assignments	169 (84.5)	29 (14.5)	2 (1)	0	3.84 ± 0.398	
	Heavy admission and discharge activity	95 (47.5)	83 (41.5)	22 (11)	0	3.37 ± 0.674	

Material factors on a scale of 3–12	Medications were not available when needed	97 (48.5)	83 (41.5)	22 (11)	2 (1)	3.32 ± 0.761	9.73 ± 1.91
	Supplies/equipment not available when needed	80 (40)	84 (42)	34 (17)	2 (1)	3.21 ± 0.754	
	Supplies/equipment not functioning properly when needed	92 (46)	62 (31)	42 (21)	2 (1)	3.21 ± 0.842	

Communication factors On a scale of 8–32	Inadequate hand‐off from previous shift or sending unit	43 (21.5)	98 (42)	55 (27.5)	4 (2)	2.90 ± 0.750	24.05 ± 4.96
	Other department did not provide the care needed	50 (25)	106 (53)	40 (20)	4 (2)	3.01 ± 0.730	
	Lock of backup support from team member	92 (46)	74 (37)	27 (13.5)	7 (3.5)	3.26 ± 0.821	
	Tension or communication breakdown with support departments	82 (41)	75 (37.5)	34 (17)	9 (4.50	3.15 ± 0.861	
	Tension or communication breakdown within in nursing team	57 (28.5)	93 (46.5)	34 (17)	11 (5.5)	2.93 ± 0.948	
	Tension or communication breakdown with the medical staff	56 (28)	87 (43.5)	41 (20.5)	11 (5.5)	2.89 ± 0.960	
	Nursing staff did not communicate that care was not provided	47 (23.5)	103 (51.5)	39 (19.5)	11 (5.5)	2.93 ± 0.805	
	Caregiver off unit or unavailable	44 (22)	114 (57)	38 (19)	4 (2)	2.99 ± 0.702	

*Note:* Item scores: 1 = not a reason to 4 = significant reason; area scores are the sum of item scores within each domain.

Stepwise linear regression showed that three communication, human, and material factors explained 85.3%, 10.2%, and 4.5% of the changes in the importance score of reasons for missed nursing care, respectively.

## 4. Discussion

This study examined the PIRNCA, as well as its causes and predictors during the COVID‐19 pandemic in Iran. The findings indicated that the RONC level was below average. These results are consistent with several previous studies. For instance, Labrague et al. reported that modifying elements of the workplace, including nurse staffing levels, safety culture, and adequacy of protective equipment, helped reduce missed care during the pandemic [32]. In Sweden, Vogelsang et al. reported that the level of missed care during the pandemic was comparable to that in normal circumstances, largely due to maintaining nurse‐to‐patient ratios and the proactive role of nursing managers in planning for the crisis [19]. Similarly, another study revealed that the rate of incomplete care during the pandemic was similar to that before the outbreak, suggesting that nurses continued to apply consistent prioritization strategies even under challenging conditions [1].

In contrast, some previous studies reported higher levels of rationing. For example, Marková and Jarošová reported that the rate of RONC in 11 hospitals in the Czech Republic was above average during the COVID‐19 pandemic in 2020–2021 [33]. Another study also indicated that the level of missed care increased during the COVID‐19 pandemic in 2020, and insufficient nursing staff was identified as the main reason for this missed care [18]. Hosseini et al. also reported that nurses in Iran faced an unfamiliar situation, which significantly affected the amount of missed care; urgent patient needs, staff shortages, and the sudden rise in patient numbers were the main causes [15]. The difference in the results of the studies can be attributed to the timing of the present study, which was conducted after the initial peaks of the pandemic. During this period, increased personnel preparedness and improved planning in response to the COVID‐19 crisis likely contributed to the observed outcomes. Furthermore, the delegation of responsibilities to diverse groups, including volunteer forces, may have played an important role in reducing the perceived RONC.

Based on our findings, the first factor—patient examination and order execution—and the second factor—documentation and support—made the greatest contributions to explaining variations in the overall care rationing score. Falk et al. also showed that most of the missed nursing care during the COVID‐19 pandemic was related to providing primary care to patients [20]. In Iran, Hosseini et al. identified that supportive and essential care were the most rationed types [15]. Labrague et al. also found that “adequate patient examination and monitoring” was the most frequently missed nursing care during the COVID‐19 pandemic [32]. These findings collectively suggest that during the pandemic, high workload and time constraints led nurses to focus primarily on essential care while postponing or omitting nonurgent tasks.

Documentation and supportive care were also notably rationed during COVID‐19. Previous studies in Iran reported that psychological and spiritual support were among the most frequently neglected aspects of care [34, 35]. Hulob et al. also noted that during the COVID‐19 pandemic, nurses tried to save time by reducing documentation and devoting more time to clinical care [36]. Moy et al. also found that throughout the COVID‐19 pandemic, significant strategies were implemented by healthcare systems at national and local levels to reduce clinical documentation, resulting in a decrease in nurse documentation and a greater focus on clinical care [37]. Overall, data documentation appears to have been significantly reduced as a result of high workloads and limited resources. Additionally, supportive care—including emotional and psychological support, an integral component of nursing practice—received less attention, likely due to the fatigue and stress experienced during critical situations.

Based on our results, several demographic and occupational variables—including interest in the nursing profession, having a second job (non‐nursing activity), number of children, and monthly income—were found to influence the RONC. As predicted, the participants interested in the nursing profession and having more children, as well as those with no second job and lower monthly income, were less likely to have RONC.

Mersin et al. similarly reported that compassionate love decreases burnout in nurses and enhances their professional commitment to providing complete care [38]. Compassionate love seems to increase the ability of nurses to provide optimal and effective nursing care, reducing the RONC. It has also been noted by Mersin et al. that nurses with children obtained higher scores on the Compassionate Love Scale and were better able to provide quality care [38].

Another study has found that the nursing profession is based on care, and care is a combination of love and respect for others. Love in the nursing profession goes beyond the traditional role of nurses, leading to integrated, patient‐centered care and greater commitment of nurses. Care, accompanied by compassionate love, is regarded as responsibility, benevolence, attention, concern, respect, and understanding of others as human beings. This approach can enhance the quality of care and subsequently reduce the rationing of care [39].

Previous research has also shown that nurses who lack satisfaction or interest in their profession are more prone to rationing care compared to their more motivated counterparts [40, 41]. When nurses are emotionally and spiritually fulfilled and find meaning in their work, they become more responsive to patients’ needs, naturally minimizing the necessity for rationing and ensuring adherence to higher nursing‐care standards. The higher level of rationing observed among nurses with higher salaries may be related to heavier workloads and broader managerial responsibilities. Better‐paid nurses often manage larger patient groups or hold administrative roles that increase time pressure, resulting in less direct patient care. Longer working hours and job‐related stress may also contribute to a greater tendency toward rationing. Consistent with these interpretations, earlier studies have demonstrated that overtime and extended work shifts contribute to fatigue and increased workload among nurses, which in turn elevate the likelihood of missed or rationed care [42, 43].

The significance of the underlying causes of nursing‐care rationing was highlighted by the high importance scores for communication and human factors. Communication factors ranked first, followed by human factors. This finding aligns with several prior studies. Khrais et al. indicated that during the COVID‐19 pandemic, the primary reasons for missed nursing care were communication issues, followed by human resource challenges and then material resource limitations [24]. Another study also found that during the pandemic, nurses face unfamiliar and critical work environments due to the crisis, high workload, changes in hospital layout, exposure to disease, and being immediately assigned to different wards without the necessary skills. In these circumstances, inadequate communication, combined with the aforementioned factors, contributes to the increased RONC [1].

A review by Papastavrou et al. [44] also identified high workload and poor communication as the major reasons for rationing, noting ineffective delegation and lack of teamwork as key communication barriers. Likewise, several studies have highlighted that human‐resource shortages and staff‐related factors are major contributors to rationed and missed care. For example, Hosseini et al. [15] found that urgent patient situations, insufficient staffing, and unexpected surges in patient volume were the most common causes of missed care during the COVID‐19 crisis in Iran. Similarly, another study reported that staffing shortages and emergency patient demands were the most frequently cited reasons for missed nursing care [20]. Therefore, both communication and human factors play a critical role in determining the extent of nursing‐care rationing.

Given the current findings regarding the perceived level of care rationing, it is essential that healthcare organizations and policymakers translate these results into actionable strategies, especially in the context of ongoing or future crises. Our findings underscore the critical need for improved staffing models and more robust communication systems within acute care settings. Policy decisions should mandate regular, objective assessments of nurse‐to‐patient ratios and ensure adequate provision of resources beyond the initial crisis phase. Furthermore, the identified contribution of communication barriers suggests that mandatory, context‐specific training on crisis communication. Finally, this study highlights the value of ongoing monitoring of rationing during crises, not just as a measure of safety, but as an indicator of workforce sustainability. Systemic monitoring allows for proactive adjustments to staffing and operational procedures before rationing escalates to unsafe levels.

While this study provides valuable insight into care rationing during the pandemic, several avenues for future research are necessary to build a more comprehensive evidence base. First, there is a significant need for longitudinal designs to track how these rationing practices evolve over time, moving beyond a single time point, particularly as healthcare systems normalize postpandemic. Second, future studies should strive to incorporate more objective performance indicators alongside self‐report measures to mitigate potential biases inherent in retrospective or stress‐influenced data collection. This could involve integrating administrative data or direct observation methods. Third, to deeply explore the complex psychological processes underpinning prioritization and rationing decisions, qualitative research designs are highly recommended to explore the lived experiences and rationale behind nurses’ rationing processes.

### 4.1. Strengths and Limitations

Among the strengths of the current study were the application of comprehensive multivariate statistical analysis, sample diversity, and stratified sampling in both hospitals, which had been facing a high number of COVID‐19 patients. This robust methodology allows for a more reliable estimation of the predictors.

However, several limitations should be acknowledged. Important factors such as hospital type and conditions, management systems, monitoring and evaluation processes, the quality of nursing care [45], and nurses’ psychological characteristics [46] were not included, despite their potential influence on care rationing. The magnitude of the linear regression coefficients also supports this notion, indicating that additional unmeasured variables may substantially contribute to the prediction of rationing scores.

Furthermore, the cross‐sectional method of this study limits the ability to establish definitive causality between the identified predictors and the level of care rationing. The reliance on self‐report measures for a sensitive topic such as the RONC may also introduce a degree of social desirability bias, potentially underestimating the true extent of missed care.

To better understand the determinants of nursing‐care rationing—particularly during crises such as pandemics—future research should employ qualitative approaches to explore nurses’ experiences in greater depth. Additionally, future studies should focus on developing and validating standardized, objective measures of care rationing across different health systems and should examine the feasibility of conducting international comparative studies to situate the Iranian findings within a broader global context of crisis response and nursing care delivery.

## 5. Conclusion

This study investigated the phenomenon of nursing care rationing in Iranian hospitals during the later stages of the COVID‐19 pandemic. Our findings revealed that the overall level of care rationing was not significantly elevated across participating institutions affiliated with the designated university of medical sciences. Key demographic and professional factors showed significant associations with care rationing: a strong interest in the nursing profession and having more children were inversely related to rationing scores, indicating a protective effect. Conversely, higher average monthly income and engagement in non‐nursing employment (a second job) were associated with higher reported rationing scores.

Critically, our analysis identified communication‐related factors as the most significant contributors to the variance in perceived importance scores for the causes of missed care. Therefore, to mitigate care rationing in future challenging situations, such as pandemics, interventions must strategically target these findings. Specifically, prioritizing the recruitment and retention of nurses with genuine professional interest, discouraging reliance on secondary employment, and strongly emphasizing the crucial role of professional communication are essential policy and practice considerations.

## Funding

This article is based on the findings of a research project approved and supported by Kashan University of Medical Sciences (Grant no. 40092).

## Conflicts of Interest

The authors declare no conflicts of interest.

## Data Availability

The data that support the findings of this study are available on request from the corresponding author. The data are not publicly available due to privacy or ethical restrictions.
